# Utilization of Site-Specific Recombination in Biopharmaceutical Production

**DOI:** 10.7508/ibj.2016.02.001

**Published:** 2016-04

**Authors:** Maryam Ahmadi, Narges Damavandi, Mohammad Reza Akbari, Fatemeh Davami

**Affiliations:** 1 Dept. of Medical Biotechnology, Semnan University of Medical Sciences, Semnan, Iran;; 2 Biotechnology Research Center**, **Pasteur Institute of Iran, Tehran, Iran;; 3 Dept. of Biotechnology, College of Science, University of Tehran, Tehran, Iran

## Abstract

Mammalian expression systems, due to their capacity in post-translational modification, are preferred systems for biopharmaceutical protein production. Several recombinant protein systems have been introduced to the market, most of which are under clinical development. In spite of significant improvements such as cell line engineering, introducing novel expression methods, gene silencing and process development, expression level is unpredictable and unstable because of the random location of integration in the genome. Site-specific recombination techniques are capable of producing stable and high producer clonal cells; therefore, they are gaining more importance in the biopharmaceutical production. Site-specific recombination methods increase the recombinant protein production by specifically inserting a vector at a locus with specific expression trait. The present review focused on the latest developments in site-specific recombination techniques, their specific features and comparisons.

## INTRODUCTION

The isolation of high-producing Chinese hamster ovary (CHO) clones for biopharmaceutical products is an industrial problem. Suitable stable cell lines are widely generated for high-level expression of recombinant proteins using random integration linking genomic amplification; however, it is very laborious and time-consuming and needs improvement. Also, due to position effects, it results in low and unpredictable yields of expression^[^^1^^]^. High-throughput selection methods, such as fluorescence-activated cell sorting or automated colony pickers, normally use some expensive procedures based on fluorescence. Vector engineering and site-specific recombination have shown acceptable results in isolating high-producing clones^[^^2^^]^. The improvement of impressive and safe non-viral vectors would considerably facilitate the complexity of recombinant protein expression. Site-specific recombination integrates transferred gene into a site with the desired surrounding chromatin. There are several commercially available systems for site-specific integration as follows: 1) two groups of recombinase family that include the tyrosine recombinase and serine recombinase^[^^3^^]^. Both families direct recombination between two recombinase target sites and result in sequence-specific DNA insertions. Since 1990s, tyrosine recombinases, such as Cre and Flp, have been used for site-specific integration in animal cells^[^^4^^]^. According to the reports, these particular recombinases can cause chromosomal aberrations. Because of the presence of pseudo-sites, control of inaccurate effects is less powerful^[^^5^^]^. Different attempts have been made to develop the efficiency and specificity of this system by redesigning their site specificity^[^^6^^,^^7^^]^. 2) Class II transposable elements, including the *Sleeping Beauty* (SB), *piggyBac* (PB), and Tol2 transposons that move in the host genome via a “cut and paste” mechanism. They account the most useful tools for genetic engineering because of their easy laboratory handling and controllable nature.

These strategies can increase biopharmaceutical protein titer and decrease the required time to achieve sufficient amounts of protein for pre-clinical evaluation^[^^8^^]^. Use of a site-specific recombinase may be expensive, and initial screening for amplifiable sites is necessary to be performed. This step is performed only once to produce an isogenic cell line that can be utilized to produce a range of desired gene products. The reviewed methods have also obvious positive effects on cell-specific productivity.

**Transposases**

**Fig. 1:**
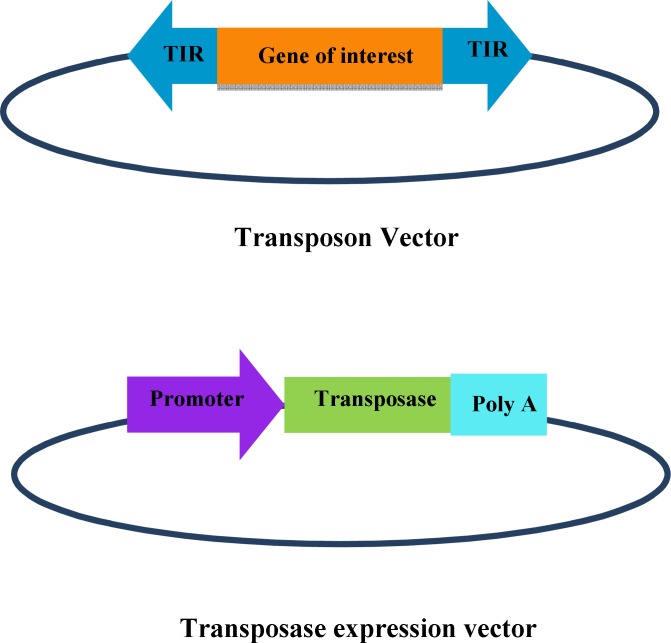
DNA transposon system. The transposon vector includes the DNA of interest flanked by transposon inverted terminal repeat sequences, and the transposase expression vector composed the transposase gene placed the downstream of a suitable promoter

DNA transposons are natural genetic elements moving via a conservative cut and paste mechanism from one chromosomal location to another. They are composed of an open reading frame coding for the transposase and flanked by two inverted terminal repeats. The molecules that support the integrative process are relatively easy for engineers since only two separate plasmids are co-delivered to the cells (Fig. 1). The donor plasmid carries transposon in which the original transposase gene is replaced with a transgene driven by an appropriate promoter, and helper plasmid carries the transposase expression cassette. Alternatively, the transgene and transposase expression cassettes can be placed on the same plasmid to simplify the process. DNA transposon has several advantages that make it a very promising tool for a wide variety of genomic methods^[^^9^^]^.

SB is a non-viral element that can integrate efficiently into the mammalian host chromosomes. The transposition of SB element always occurs into a TA target dinucleotide, which is subsequently duplicated upon insertion by cellular DNA repair pathways^[^^10^^]^. SB target site selection is determined by structural constraints rather than primary DNA sequence. In contrast to most retroviral-based vectors, SB integrates somewhat randomly in mammalian cells, without any discernible preference for actively transcribed genes^[^^11^^]^. This feature makes SB a suitable candidate for development into targeted systems. The first step towards specific site integration at the genome scale was achieved in 2007 with an SB transposase fused with the E2C zinc-finger protein. However, these systems are still based on random integrations^[^^12^^]^. 

Tol2, a hAT superfamily, is another transposon system that can transfer large transgenes of up to 11 kbp with minimal loss of transposition activity^[^^13^^]^ and less preventive effect on transposition. Tol2 transposase activity is more susceptible to be affected by molecular engineering, as described for SB, and its targeting preference is not obvious. Also, 5’ regions of genes are most preferred for Tol2, the same as other hAT elements^[^^14^^]^.

PB, a superfamily of PB, is a DNA transposon isolated from the cabbage looper moth. It integrates into TTAA sequences, and transposition occurs via a cut and paste mechanism in which the PB transposases are initially recognized and bind to the transposon termini^[^^10^^]^. It then excises the entire transposon from its original location and catalyzes its integration at another chromosomal site through a mechanism that is not dependent on host factors^[^^11^^]^. In comparison, PB is an alternative to SB and even to Tol2 because of its large cargo size (up to 14 kb), high activity in many cell types, long-term expression in mammalian cells, less susceptibility to be affected by molecular engineering, and the ability to excise precisely^[^^15^^]^. 

PB and Tol2 have shown similar error rates in insertion into genes, especially introns or getting close to transcription start sites. There are some pieces of evidence that active chromosomal regions favor the PB and Tol2 integration processes^[^^16^^]^. It seems that host factors have an important role in PB and Tol2 integration rates. Also, the potential risk of undesirable integration cannot be excluded and the continuous expression of an inadvertent integrated transgene can result in genotoxic risk^[^^17^^]^. Transposable element, such as PB and SB, have been used for integrating recombinant genes into cultivated mammalian cells^[^^18^^-^^20^^]^. Using PB transposon, recombinant CHO cell lines are able to produce up to four-fold more recombinant protein in comparison to standard transfection and are stable up to three months in the absence of selection^[^^21^^]^. 

**Cre/loxP recombination**

**Fig. 2:**
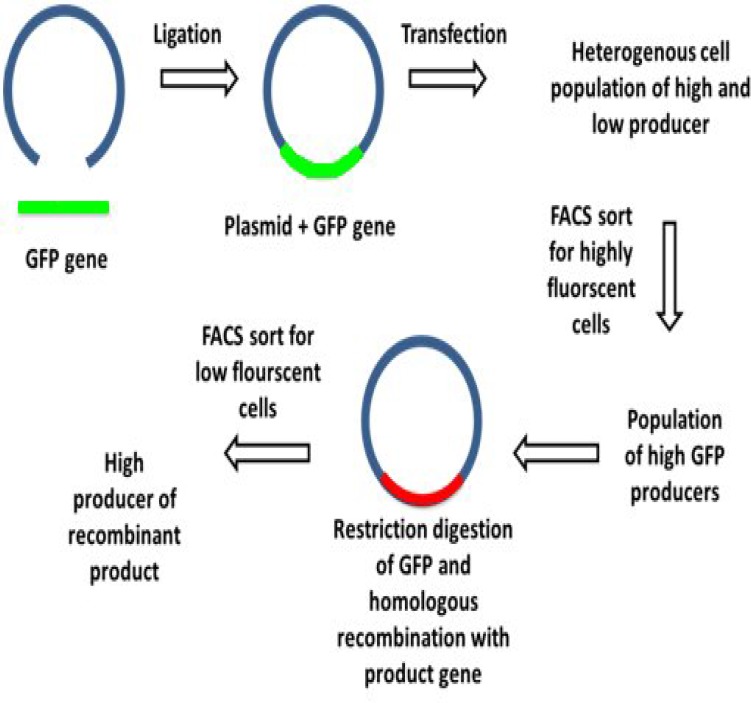
Schematic diagram of Cre/loxP recombination system

Cre/loxP recombination system is a gene-targeting method for targeting genes to specific sites of genome with suitable expression levels^[^^22^^]^. Cre recombinase recognition site, known as a loxP site, is a 34-bp DNA sequence that will remain after integration. Therefore, this reaction is reversible by the loxP site. Despite this disadvantage, the Cre/loxP system is able to generate high specificity by about 80%^[^^23^^]^. In an experiment, dhfr-deficient CHO cells were transfected with a vector containing the green fluorescent protein reporter and a *dhfr* gene downstream of loxP site^[^^22^^]^. The selection of positive clones was first based on the highest fluorescence and in the second step, methotrexate amplification-selection procedure was performed. Then, a targeting vector containing the antibody of light and heavy chain genes was fused to a hygromycin resistant marker and a loxP site was co-transfected along with Cre recombinase to catalyze the site-specific recombination. The targeting vector was integrated into a locus that was transcriptionally active and amplifiable (Fig. 2). This issue resulted in producing 160 mg/L human monoclonal antibody in seven days; a substantial improvement compared to the previously reported value of 40 mg/L obtained under similar conditions^[^^23^^]^. This method showed a noticeable increase in production titers since gene integration occurred at a location that was pre-selected for its ability to be amplified. In comparison to traditional methods, because of the elimination of the second round of amplification after establishing platform cell line, this method was less time-consuming. Also, the requirement of the Cre recombinase might cause an increase in the cost of this method. 

**Flp/FRT recombination**

 Another gene-targeting system is known as Flp/FRT by Flp recombinase and gene sequences tag with a FRT sequence. Flp/FRT system has weak recombination specificity (10%)^[^^24^^]^. Using a vector containing two weakened markers (*β-galactosidase* and *dhfr*) and an FRT sequence for subsequent recombination into the transcriptionally active sites, cells were screened for different gene integration sites. Then 20 candidate clones were selected for amplifiable expression sites, three of which were suitable as hosts for Flp recombination of antibody genes. After six days of culturing, up to 200 mg/L full-length anti-CD20 antibody was produced in a spinner flask. Other report have shown that this method can be used in a broad range of target genes^[^^25^^,^^26^^]^. In other similar works utilizing the Flp/FRT system^[^^24^^]^, a target vector was tagged by FRT and an antibiotic resistance marker. Also, high producing clones were isolated for tissue plasminogen activator, and the highest specific productivity achieved was 17.1 μg/10^6^ cells/day. Therefore, this method is very similar to the Cre/lox system with the same advantages and limitations.

Φ**C31 integrase recombination**

**Fig. 3:**
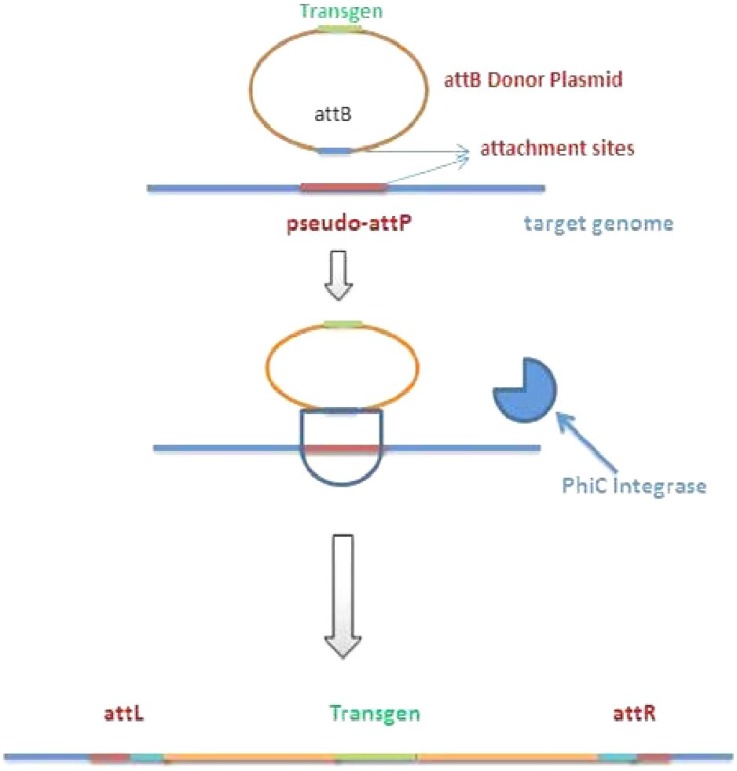
Schematic diagram of the ΦC31 integrase-mediated recombination of donor plasmid sequence into *pseudo-attP* sites in host genome.

ΦC31 is a serine recombinase from the streptomyces phage f31 that can catalyze recombination between f31 phage *attP *site and the bacterial host *attB* site. There are up to 100 target sites for this system in the human genome^[^^27^^]^; however, the selection of specific inegration sites in this system is impossible. The high rate of chromosomal translocations and the risk of gene-toxic effects such as cancer are important concerns related to ΦC31. The obvious advantage of ΦC31 integrase over Cre and FRT systems is its irreversibility. The ΦC31 integrase catalyzes recombination between *attP* and *attB* sites, which consist of different sequences. Two resulting recombination hybrid sites cannot be substrates for the integrase activity (Fig. 3). Another advantage is the presence of several *pseudo-attP* sites with good sequence similarity to native* attP *sequence in mammalian genome that can act as substrates for the enzyme. A disadvantage of ΦC31 in comparison to Cre/loxP is its low specificity that is almost 10%, similar to Flp recombinase. By protein engineering method, a mouse codon-optimized mutant of ΦC31 integrase known as ΦC31o was developed^[^^24^^]^. Recombination specificity of this mutant version of the enzyme was identical to that of Cre recombinase. CHO cells were co-transfected with a plasmid transcribing the ΦC31 enzyme and a plasmid containing an *attB* site for integration into *pseudo-attP* sites and a gene for luciferase reporter. A 60-fold higher expression yield has been reported using this recombination system compared to random transfection methods^[^^27^^]^. To take more advantage of genome engineering capacities of ΦC31 integrase, it can be combined with other site-specific recombinases that are highly specific but recognize only their own *attP *sites (not *pseudo-attP *sites)^[^^28^^]^. There are many integrases that are functional in mammalian cells and have been commonly used in combinatorial systems, such as Bxb1, R4 Cre, and Flp (Table 1). 

**Artificial chromosomes expression system (ACE)**

**Fig. 4:**
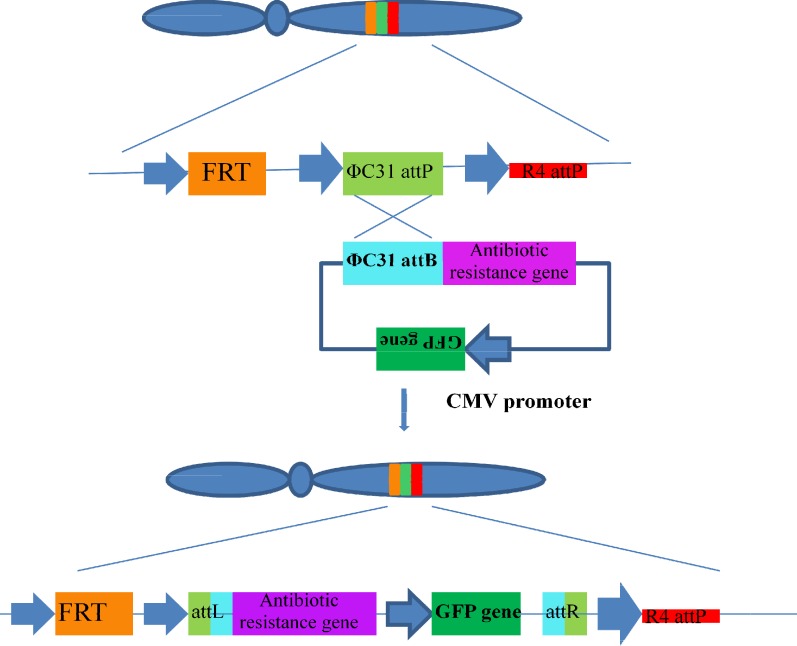
Diagram of the insertion of the plasmid containing the cytomegalovirus promoter and the green fluorescent protein gene in the multi-integrase human artificial chromosomes HAC vector or host chromosome

Human artificial chromosomes have several advantages over conventional gene delivery systems and seem highly promising^[^^29^^,^^30^^]^. Artificial ACE is composed of a mammalian based artificial chromosome (Platform ACE), an ACE-targeting vector, and a mutant λ integrase (ACE integrase) for site-specific recombination^[^^31^^,^^32^^]^. The platform ACE consists of tandem repeated ribosomal genes, repetitive satellite sequences (from the pericentromeric heterochromatin), natural centromeres, and telomeres to enable DNA replication without need for integration into the host cell genome, which reduces clonal heterogeneity because of chromosomal aberration. Recombination acceptor of *attP* sites in platform ACE is about 50–70, thus allowing for the incorporation of multiple copies of the considered gene. The platform ACE cell line is cotransfected with the ACE-targeting vector and ACE integrase plasmid. Following the recombination event, promoterless selection marker on the ACE-targeting vector integrates the downstream of the SV40 promoter in the platform ACE. Therefore, under selection pressure, the survived cells are identified as clones that have undergone a correct recombination event (Fig. 4). Utilizing this approach, high-expressing clones select from 100 to 200 cell clones, and yield of monoclonal antibodies expressing cell line by this system has been more than 500 mg/L in batch terminal shake flask cultures^[^^33^^]^.

**Gene targeting in applications other than protein expression**

Transposable elements are important tools for insertional mutagenesis and transgenesis^[^^34^^]^. For genome manipulations in vertebrates and mammals, the transposon-based tool was started by SB reactivation in 1997. The SB has been successfully used for genetic modifications of human cell lines and various vertebrates^[^^13^^,^^35^^,^^36^^]^. The PB system has been applied for different applications such as germline or somatic mutagenesis, gene therapy, and gene transfer in mammals or human cells^[^^37^^]^. The PB vector has been used to produce induced pluripotent stem cells^[^^38^^]^. SB and PB systems have been applied for the modification of CD34+ hematopoietic stem cells^[^^39^^,^^40^^]^.

**Table 1:** Brief comparison of transposase, recombinase, and integrase approaches

Approach	Advantage	Disadvantage
**Transposases** *** Sleeping beauty*** *** piggyBac***	*piggyBac*	Reversible
		
**Tyrosine recombinases Cre/loxP ** ** Flp/FRT**	Less or no Amplification	Reversible
		
**Serine integrases** ** Φ** **C31**	Irreversible integration in transcriptionally active the part of genome Stability Less or no amplification	Amplification is required for high-level expression

The site-specific recombinases have been proven to be useful tools for the analysis of gene function both *in vitro* and *in vivo*^[^^41^^,^^42^^]^. Transgenes integration into human embryonic stem cells has been also reported using ΦC31 system technique^[^^43^^]^. Because of genotoxicity, ΦC31 system does not seem suitable in human gene therapy applications^ [^^44^^]^. Artificial chromosomes and minichromosome-like episomes are very promising tools for gene therapy of inherited diseases caused by recessive mutations such as hemophilia or Friedreich's ataxia^[^^45^^]^.

**Other gene-targeting approaches in biopharma-ceutical production**

There are a number of methods rather than site-specific recombination for cell line engineering and biopharmaceutical purposes. Below is a summary of the high-impact approaches:

Lentiviral vectors integrate into the host genome of both dividing and non-dividing cells. These complex retroviruses can be used to express recombinant proteins in various cell types^[^^46^^,^^47^^]^.

The lentiviral system is naturally flexible and modular and allows for the insertion of nucleic acid sequences fewer than 6 kb in length^[^^48^^]^. Lentiviral vectors could generate high producer clones expressing recombinant proteins such as blood coagulation factor VIII, secreted alkaline phosphatase^[^^49^^]^, and tumor necrosis factor, Fc fusion protein^[^^50^^]^, even without chemical selection in serum-free media. Until now, several approaches, including fusion of viral integrase to a hetrologous DNA-binding domain protein^[^^51^^,^^52^^]^ and combining site-specific recombinase systems with lentiviral vectors^[^^53^^]^, have been invented to mediate lentiviral integration in a site-specific manner. However, the integration of retroviral DNA is heavily biased towards trans-criptionally active genes, which could compromise the potential utility of any directed integration strategy^[^^54^^]^.

Zinc-finger nucleases and transcription activator-like effecter nucleases comprise a powerful class of tools for biological research. These chimeric nucleases are composed of programmable, sequence-specific DNA-binding modules linked to a non-specific DNA cleavage domain^[^^55^^]^. Zinc-finger nucleases and transcription activator-like effecter nucleases enable a broad range of genetic modifications; however, cell line development using DSB-mediated targeted integration has been reported only in a very few cases^[^^56^^]^, probably due to intellectual property obstacles^[^^57^^]^.

The clustered regularly interspaced short palindromic repeats (CRISPRs)/Cas system, which uses re-programmable trans-activating CRISPR-RNA for sequence-specific cleavage, has emerged as an efficient tool for genomic modifications^[^^58^^]^. The system has been successfully used for genome editing in CHO for biopharmaceutical development^[^^59^^,^^60^^]^. Using this system, the site-specific integration of the therapeutic protein gene in CHO has been reported^[^^61^^]^. However, additional studies are required to evaluate the specificity and the toxicity of RNA-guided DNA endonucleases.

Mammalian cell protein expression system has been the dominant recombinant protein production system for clinical applications in last two decades. This system has produced more than half of the biopharmaceutical products in the market and several hundreds of candidates in clinical development. Furthermore, many improvements have been made in cell line engineering methods, genetic methods of expression, gene silencing, and gene-targeting systems. Due to inefficiencies of traditional random integration methods in producing appropriate yields expression in industrial scales^[^^62^^-^^65^^]^, numerous studies are nowadays dedicated to establishing efficient, targeted gene integration systems. There are different enzymes that have to be engineered to induce and target a double-strand break for recombination. 

New site-specific non-viral vectors based on a given recombinase, integrase, or modified transposase can direct integration into a related site with a similar nucleotide sequence and thus correct random integration limitations and immunogenicity in gene therapy vectors.

Transposon-based vectors, such as SB, PB, and *Tol2*, increase integrated gene copy numbers and improve the recombinant protein titer as compared to the standard transfections^[^^66^^]^. However, the random inherent of transposases is a major drawback for their genomic modification applications. Phage recom-binases (Cre, Flp, and ΦC31) enable an efficient and a site-specific integration of transgene and are more easily vectorizable. The *in vitro* GATEWAY^TM^ cloning method (invented by Invitrogen^TM^ USA) with an impact on a variety of research areas benefits from phage integrases^[^^67^^]^. The site-specific non-viral vectors based on the ΦC31 integrase have been promising in achieving long-term therapeutic gene expression through integration in tissue culture cells and in animals. However, these systems are limited by the need for a prior establishment of platform cell lines and the possibility of chromosomal aberrations^[^^68^^]^. The artificial engineered mammalian chromosomes are an example for an ideal gene delivery vector, with a stable episomal maintenance and a large transgene capacity. However, some factors, such as having a fragile construct, difficulties for purification, and low transduction efficacy, limit the application of human artificial chromosomes^[^^69^^]^. There is still much need for efforts to further improve the methods for designing recombinases with altered specificity and with combinatorial structures that would be more practical. Therapeutic potential of targeted recombination systems and optimal methods for delivering these enzymes into different cell types are the other fields that need continued exploration. 

In addition to above mentioned methods, the recent tool CRISPR/Cas could be an advantageous choice for the future, but features such as off-target mutations and unwanted chromosomal translocations associated with off-target DNA cleavages^[^^70^^]^ also represent challenges for this system. The latest improvement of gene-targeting methods for mammalian recombinant proteins expression, as an important subcategory of cell line development, was summarized in this review. 
